# News and Events of the Week

**Published:** 1910-08-13

**Authors:** 


### News and Events of the Week.

The Royal Humane Society's Medal has been awarded to Nurse Eva Lindsay, of the Military Hospital, Dundee, who attempted to rescue a lady from drowning.

Mr. David Davies, L.S.A., L.R.C.P., F.R.C.S., of Bryngolwg, Aberdare, Glamorgan, who died on March 17, aged eighty-seven, left estate valued at £82,514 gross, with net personalty £80,867.

Jeyes' Fluid has been awarded the Grand Prix—the highest possible award—at the Japanese-British Exhibition. This is the 133rd gold medal or other award which this famous disinfectant has secured on such public occasions.

An inquest was recently held on the body of a man who, while on a holiday in the country, was stung in the forehead by a bee, while gathering mushrooms. He died half an hour later. A medical examination showed that death was due to shock.

Sir William Treloar announces that among other contributions to the fund he is raising on behalf of the widow of the late Mr. H. W. Cox, the *x*-ray martyr, he has received £150 from his Majesty's Royal Bounty, upon the recommendation of the Prime Minister.

A public meeting was held at Derby this week under the presidency of the Mayor in order to form a local branch of the British Red Cross Society. The Duke of Devonshire, dealing with the relations between this society and the St. John Ambulance Association, said it was preposterous that there should be any wrangling or quarrelling upon this subject. He hoped the movement would be cordially supported in Derbyshire.

Dr. George Oldham Siddall, M.R.C.S., L.S.A., of The Lockyer Hotel, Plymouth, for some years proprietor of that hotel (and of Alfreton, Derbyshire), who volunteered for medical service during the Baltic war and served on board the *Arrogant* in 1855, and was present at the boat action at Viborg and the bombardment of Sveaborg, died on June 1, aged seventy-five, and left estate valued at £18,347 gross, with net personalty £18,234.

The Finsbury Borough Council has granted a contract for ten years to the Gas Light and Coke Company for the lighting of all the borough streets by new inverted burner lamps, which will show an increase in total illuminating power of 100,000 candle power over the existing system of lighting, but with a reduction in cost of £525 a year. This decision was taken after the latest examples of gas and electric lighting had been carefully inspected and their relative costs considered.

We have received from the Funk and Wagnalls Company, of Salisbury Square, E.C., a supplementary list of about 500 recent words which have been added to the vocabulary of their standard dictionary. Many of the words are in use in medicine and the allied sciences. Pharmacy is represented by definitions of adrenalin, aspirin, dionin, dormiol, formin, pinene, and veronal. A number of medical terms are defined, such as colpeurynter, dhobee-itch, hypaesthesia, masochism, megaloblast, opsonin, pediatrist, psychasthenia, and stegomyia. Ophthalmology has necessitated the addition of definitions of erythropsia, esophoria, hyperphoria, hypophoria, ligne, and retinoscope. The ever-growing language of "slang" accounts for the addition of many words.

The following are the arrangements for next week at the West London Post-graduate College, West London Hospital, Hammersmith Road: August 15 and 18, Demonstrations by the Surgical Registrar at 10 a.m.; August 15, Pathological Demonstration, at 12 noon; August 16, Lecture by Dr. Bernstein on "Laboratory Aids to Diagnosis," at 5 p.m.; August 17, Lecture by Dr. Grainger Stewart, on "Practical Medicine," at 12.15 p.m.; and August 19, Lecture by Mr. Bishop Harman, on "Corneal Ulcers," at 5 p.m.

The winter session of St. George's Hospital Medical School (University of London), Hyde Park Corner, S.W., will open on Saturday, October 1, when the prizes will be distributed and the annual oration delivered by Dr. S. Squire Sprigge, editor of the *Lancet*, at three o'clock. The title of the oration will be "On Prizes." At 4.15 the annual meeting of the St. George's Hospital Club will take place. The annual dinner will be held at Prince's Restaurant at 6.30 for seven o'clock, G. R. Turner, Esq., F.R.C.S., surgeon to the hospital, in the chair. Applications for dinner tickets should be addressed to "The Dinner Secretaries, St. George's Hospital, S.W."

The awards at the Japan-British Exhibition have just been published, and we notice that "Lemco" and "Oxo" have again obtained the highest possible honour, as in 1908 and 1909, viz., the Grand Prix. The success of this great company (the largest in the world devoted solely to the manufacture of concentrated beef foods) has been phenomenal since its formation forty-five years ago, when it was awarded a gold medal at the first great Paris Exhibition of 1867 for founding a new industry. Captain Scott's ship, the *Terra Nova*, carries large supplies of the company's products for use in the Antarctic. In this connection our readers will probably remember Lieutenant Sir E. H. Shackleton's historic cable on his return to New Zealand from the Antarctic, that he had "found 'Oxo' excellent in sledge journeys and throughout the winter."

On a motion for stay of proceedings and compensation under the Workmen's Compensation Act an application was made last week in the Westminster County Court on the ground that the injured workman Perry had refused to submit to a medical examination. On behalf of Perry it was denied that there had been any refusal to submit to a fair examination. There had been an offer to submit to the examination with the condition that it should be at the man's solicitors' office if Dr. Collie conducted it, and in the presence of the man's legal representative. This condition, it was contended, was reasonable, as it was alleged that Dr. Collie behaved with brutality and called those he examined malingerers and lazy men. The solicitor for Perry said that for some years he had objected to Dr. Collie on account of the way he treated men, shouting at them and frightening them so that they could not tell what they were suffering from. The workman said Dr. Collie told him not to wear the bandage he had on his leg, and made him walk without his stick. Dr. Collie, in answer to the judge, said he found the presence of a layman at an examination was embarrassing and sometimes nullified the whole of the doctor's work. Judge Woodfall said reason had been given by the workman for refusing to be examined in the presence of another medical man. He thought it preposterous to say a layman should be present at a medical examination. The application would be granted.

### DEATH UNDER ANÆSTHETIC.

An inquest was held by the City Coroner in his Southwark Court on Friday, July 29, 1910, relative to the death of a tramcar driver, who died in Guy's Hospital under ether, given for the purposes of an operation. Dr. Waldo, on opening the case, told the jury that the house-surgeon who gave the anæsthetic had written to him and offered to certify the cause of death as a natural one from peritonitis. He (the coroner), however, was obliged, under the provisions of the Coroners' Act, to hold public inquiries on all cases reported to him where there was a reasonable presumption that the death was a violent or unnatural one; and in all such cases it was his custom to order an autopsy to be made by an independent skilled pathologist. The widow stated that on the 2nd inst. her husband, who was a strong, healthy and sober man, after partaking of fried fish and chips the previous day, had complained of pain in his stomach. A local doctor, who was called in, sent him to Guy's Hospital, where he arrived the same day at 4 P.M. Not being satisfied with the stated cause of death she had asked the coroner to hold an inquest. The house-surgeon at Guy's Hospital said the case was one for immediate operation, and gave ether by the "open" method. Five minutes later the patient died suddenly, the abdomen having been shaved preparatory to a section for appendicitis. Asked by the coroner why the giving of the anæsthetic had not been undertaken by one of the ten visiting special anæsthetists at Guy's Hospital considering the fact that the patient was suffering from frequent vomiting and the seriousness of the case, the witness said the patient was prepared for the operation at 6 P.M., by which time all the specialists had left the hospital. The acting resident surgeon at Guy's Hospital said he did not think the case required any very special skill, and the previous witness was a competent anæsthetist. The visiting anæsthetists at Guy's Hospital were looked upon more as teachers than as actual administrators of anæsthetics. The coroner remarked that with regard to Guy's Hospital he noticed that in nearly every one of the forty-four cases of those reported as having died under anæsthetics during the past nine years—on all of which he had held public inquests—the anæsthetic had been given by junior residents. In another great general hospital during the same period where it was the custom for the anæsthetic in serious cases to be given by visiting and resident specialists there had been fewer fatalities, and very few indeed, not more than two or three in number, at the hands of the junior residents. In difficult cases he could not help feeling that it would be better if anæsthetics were administered either by seniors, or if by juniors at least in the presence and under the guidance of specialists. In October 1907 a Southwark jury, inquiring into the death of a young married woman (Mrs. Morgan) who died under chloroform given by a junior resident for an operation for the cure of exophthalmic goître, recommended this reform to the authorities at Guy's Hospital, also that in future full details of all administrations of anæsthetics should be kept in proper registers by the hospital authority. In reply, Mr. Hughes said that the jurors' rider in question had resulted in a full inquiry by the hospital authority. As a practical outcome of such inquiry the four following reforms had since been introduced: (1) The appointment of two additional teaching visiting special anæsthetists, now making ten in all at the hospital. (2) The requirements that all candidates should before appointment on the junior staff produce evidence of special practical knowledge in the administration of anæsthetics. (3) The keeping of a full register of all administrations of anæsthetics. (4) The introduction of the administration of ether by the "open" method, such as was in use on its first discovery and at the present time largely practised in the United States of America.

The pathologist who had made an autopsy in the presence of the anæsthetist, said death was due, in his opinion, to the rupture of a small ulcer in the intestine which had led to peritonitis. This had caused vomited matter to regurgitate and lodge in the air-passages whilst the patient was under the influence of the anæsthetic, thus producing suffocation. He did not think death had been hastened by the ether. In any case the patient would probably have died within a short time as a result of the peritonitis. He could not say whether or not the fried fish and potatoes or other food taken by the deceased had any connection with the fatal result. In answer to the coroner, the witness said they had a resident special anæsthetist at St. Mary's Hospital, which was the case also at a few of the other metropolitan hospitals.

In summing up the coroner said he felt sure all would be pleased to hear of the reforms introduced and of the decrease of late in the number of deaths under anæsthetics at Guy's Hospital. Since 1907, in which year there were eleven fatalities, the deaths reported were few and far between. Two deaths had been inquired into during the present year. In the first of these—a death under ether given by the "open" method—the verdict was to the effect that the cause of death was not in any way influenced by the anæsthetic. The second case—the one now under consideration—seemed on the evidence to be a similar one to the former death. As a result of the inquests referred to questions had from time to time been asked in the House of Commons. This had led to the late Home Secretary (Mr. Gladstone) referring the matter to the Departmental Committee appointed to inquire into the law relating to Coroners and the practice in Coroners' Courts. The first recommendation of this Committee in their report was to the effect that "every death under an anæsthetic should be reported to the coroner, who, after inquiry, should determine whether it is desirable to hold an inquest or not." With the first part of this suggestion he heartily agreed, since it was a well-known fact that only a small proportion of deaths under anæsthesia in private practice ever came to the notice of registrars or of coroners. With the latter part of the recommendations, however, he disagreed, since he did not think such a change in the law at all favourable to the public interest. He preferred the English open method of inquest by coroner and jury to the private inquiry as held by the Procurator Fiscal in Scotland into violent or unnatural deaths. Such secret investigations were, he thought, hardly favourable to a full statement of the facts with regard to so delicate a matter as that of fatal anæsthesia.

The jury returned a verdict in accordance with Dr. Spilsbury's evidence.

---

Mr. Charles Terry, of Foxhill Grove, Perrymead, Bath, retired surgeon, who died on May 22, aged eighty-five, left estate of the gross value of £53,566, of which the net personalty has been sworn at £33,274.

---

The Government has declined to take part in the International Hygiene Exhibition to be held at Dresden in 1911, considering that it would not be practicable to arrange an official exhibit, owing to the reluctance of manufacturers in this country to incur the heavy expenditure involved in frequent participation in large international exhibitions, and in view of the other engagements entered into by his Majesty's Government in respect of other exhibitions to be held during this year and next.

